# 3-Amino-8-hydroxy-4-imino-6-methyl-5-phenyl-4,5-dihydro-3*H*-chromeno [2,3-d ]pyrimidine: An Effecient Key Precursor for Novel Synthesis of Some Interesting Triazines and Triazepines as Potential Anti-Tumor Agents

**DOI:** 10.3390/molecules171011538

**Published:** 2012-09-27

**Authors:** Mohamed G. Badrey, Sobhi M. Gomha

**Affiliations:** 1 Chemistry Department, Faculty of Science, Fayoum University, El-Fayoum, 63514, Egypt; Email: mohammedgomaa2006@yahoo.com; 2 Department of Chemistry, Faculty of Science, University of Cairo, Giza, 12613, Egypt

**Keywords:** pyrimidotriazines, pyrimidotriazepinones, triazolopyrimidines, antitumor activity, hydrazonoyl halides

## Abstract

A number of interesting heterocycles were prepared through interaction of the intermediate 3-amino-8-hydroxy-4-imino-6-methyl-5-phenyl-4,5-dihydro-3*H*-chromeno-[2,3-d]pyrimidine (**1**) and reagents such as hydrazonyl halides **2** to furnish triazine derivatives **4a**–**l**. Reaction of **1** with phenacyl bromide afforded compound **5**. Moreover, the title compound **1** was subjected to condensation with active methylene compounds (ethyl acetoacetate and ethyl benzoylacetate) to give triazipinones **8a,b**. The condensation with aromatic aldehydes afforded either the triazole derivatives **10a**–**d** or Schiff base **11**. In addition, the behaviour of compound **1** towards activated unsaturated compounds namely dimethyl acetylene dicarboxylate and ethoxymethylenemalonitrile was studied and it was found to furnish the triazine **13** and triazepine derivative **15**, respectively. Combination of title compound **1** with chlorinated active methylene compounds delivered the triazine derivatives **18a**–**c**. Reaction of **1** with chloroacetonitrile furnished compound **20**. The structures of the products were elucidated based on their microanalyses and spectroscopic data. Finally, the antitumor activity of the new compounds **4a** and **8a** against human breast cell MCF-7 line and liver carcinoma cell line HepG2 were recorded.

## 1. Introduction

The word tumor is commonly used as a synonym for a neoplasm [a solid or fluid-filled (cystic) lesion that may or may not be formed by an abnormal growth of neoplastic cells] that appears enlarged in size [1]. In modern medicine, the term *tumor* means a neoplasm that has formed a lump. While cancer is by definition malignant, a tumor can be benign, pre-malignant, or malignant, or can represent a lesion without any cancerous potential whatsoever. Development of novel drugs, and in particular new antitumour agents is a constantly growing need that concerns researchers throughout the World, consequently, as cancers continue to be an emerging problem. Numerous antitumor chemical drugs have been widely synthesized, including the chromenopyrimidines, which present interesting biological activities. The authors, who have contributed in the past to the exploration of this research topic, were interested in expanding their work by developing a facile synthesis of new derivatives and then test their antimicrobial, cytotoxicity activities [1,2,3], and *in vitro* antitubercular activity [4], in addition to antitumour activity. It was reported that pyrimidotriazines themselves posses biological activities with a wide range of applications [5,6,7,8,9]. The research done in this article could be regarded as an extension to our previous work [10] for constructing fused chromenopyrimidines heterocycles through reactions of the key compound **1** with a variety of reagents, especially hydrazonyl halides [11,12,13,14], which lead to interesting azoheterocyclic compounds.

## 2. Results and Discussion

### 2.1. Chemistry

**Scheme 1:**
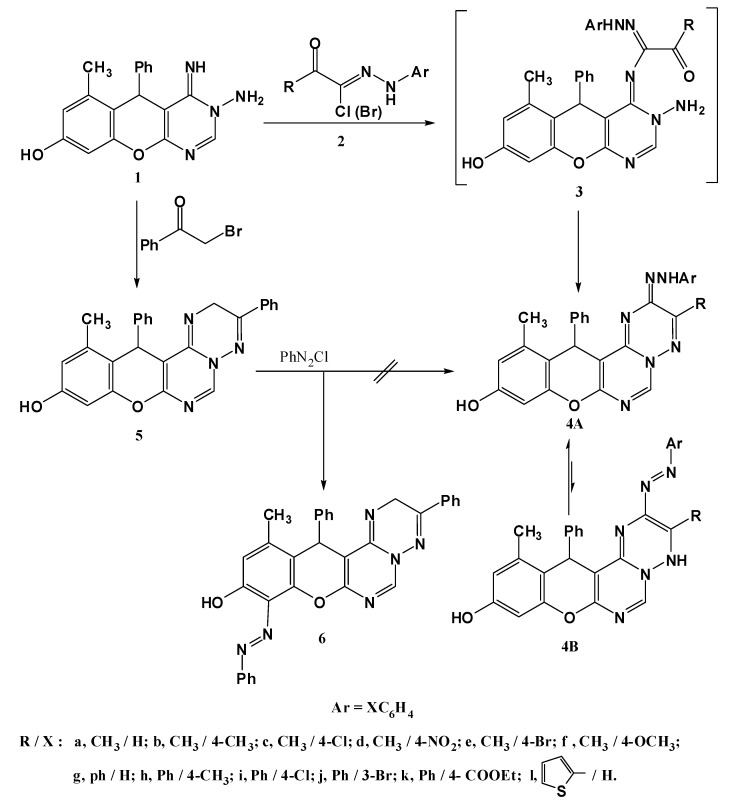
Synthesis of 5-arylazotriazine derivatives **4a**–**l**.

The title compound **1** was prepared according to the procedure reported in literature [10], and it was proved to be highly reactive towards various reagents, resulting in the formation of a wide range of annulated chromenopyrimidine systems. With compound **1** in hand, a number of valuable heterocycles could be prepared. Firstly, the interaction between the aminopyrimidine **1** and hydrazonyl halides **2** in refluxing ethanol delivered the azotriazine derivatives **4a**–**l** in good yields (Scheme 1). Structure assessment was based on their spectroscopic data. The IR spectra showed absorption bands at 3,470–3,410 (OH), 3,350–3,310 (NH) and at 1,593–1,573 cm^−1^ (C=N), while the mass spectra revealed molecular ion peaks consistent with the proposed structures. The ^1^H-NMR spectra, for example for compound **4a**, showed enrichment of the aromatic signals due to the additional aryl group, while two signals at 9.30 and 9.66 ppm for two D_2_O exchangeable protons (NH, OH) also appeared. The spectral data presented here indicate collectively that such compounds **4a**–**l** exist predominantly in the hydrazone tautomeric form **4A** rather than **4B** [15,16,17,18,19,20].

In order to prepare an authentic sample of compound **4a** through an alternative route, the aminopyrimidine **1** was condensed with phenacyl bromide **6** in ethanol to afford the triazine derivative **5**, whose structure was elucidated from its spectroscopic data. The mass spectrum showed a peak of *m/z* = 420 corresponding to the M.F. C_26_H_20_N_4_O_2_, while the ^1^H-NMR displayed a signal at 4.23 ppm attributable to a CH_2_ group. When compound **5** was coupled to phenyldiazonium chloride, unfortunately, it failed to yield the desired compound **4a** because the diazonium salt coupled preferentially to the more reactive phenolic ring to give compound **6** (Scheme 1). The structure of compound **6** was established by its spectroscopic data compatible with the proposed structure (see Experimental).

**Scheme 2:**
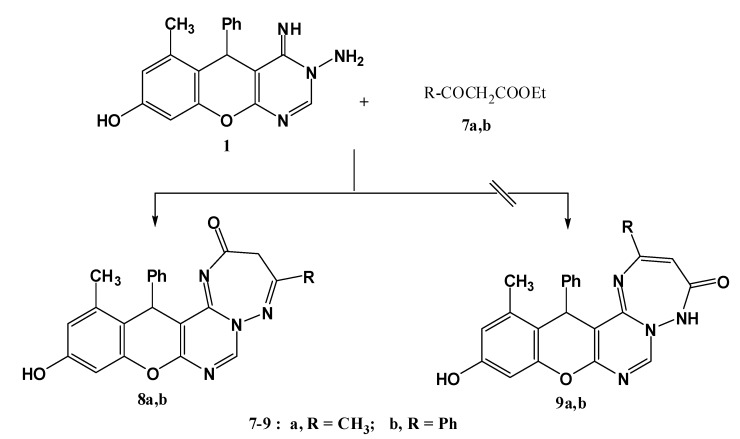
Synthesis of triazepine derivatives **8a,b**.

An even more convenient access for constructing triazepines based on the aminopyrimidine compound **1** was established using readily available active methylene reagents such as ethyl acetoacetate (**7a**) and ethyl benzoylacetate (**7b** to convert the pyrimidin-8-ol **1** into the triazepinone derivatives **8a,b** instead of **9a,b** [18,19,20,21,22,23,24,25] (Scheme 2). The mass spectra of these compounds revealed peaks at characteristic *m/z* values corresponding to their molecular weights. In the ^1^H-NMR spectra, for example R= CH_3_, a signal at 4.06 ppm integrating for two protons (CH_2_), and only one downfield characteristic signal (D_2_O exchangeable) corresponding to OH proton at 9.72 ppm, excluded the structures **9a,b** as reaction products since they lack a CH_2_ group and contain an NH function (no characteristic signal in ^1^H-NMR).

**Scheme 3:**
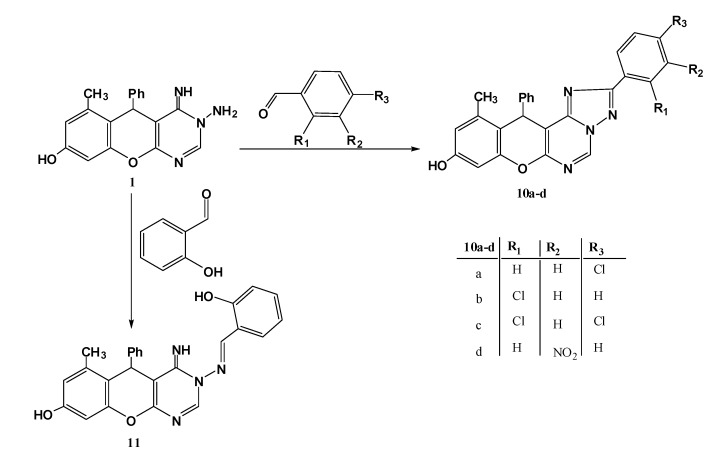
Reaction of title compound **1** with aromatic aldehydes.

In continuation to our previous work on the title compound **1** and studying its behaviour towards aromatic aldehydes via condensation in basic medium (piperidine), the aromatic aldehydes were reacted in a different manner, all of which afforded the triazole derivatives **10**, except salicyaldehyde that just gives the ordinary Schiff base **11** (Scheme 3).

It worth mention that when an EWG (Cl, NO_2_) is in *o*-, *m-* or *p*-positions with respect to the aldehydic function, a condensation followed by cyclization occurred to give the triazoles **10**, otherwise, the Schiff bases were produced, which is no doubt due to the + and –M effect of these substituents. The mass and ^1^H-NMR spectra were sufficient to indicate the correct structures, for example the mass spectrum for the reaction product obtained from reaction with *p*-chlorobenzaldehyde showed a peak at *m/z* = 440 consistent with structure **10a** (the Schiff base should give *m/z* = 442). The ^1^H-NMR spectrum revealed one downfield CH=N-signal at 9.67 ppm (the Schiff base should show two downfield signals for CH=N- protons). On the other hand, reaction of **1** with salicylaldehyde afforded the Schiff base **11**, based on its spectroscopic data; the mass spectrum showed a molecular ion peak at 424 (triazole **10** should give 422). Also, the ^1^H-NMR displayed two downfield signals at 8.34, 8.36 ppm corresponding to CH=N and 2-H protons, in addition to two D_2_O exchangeable signals (NH, OH) at 5.86 and 8.62 ppm.

**Scheme 4:**
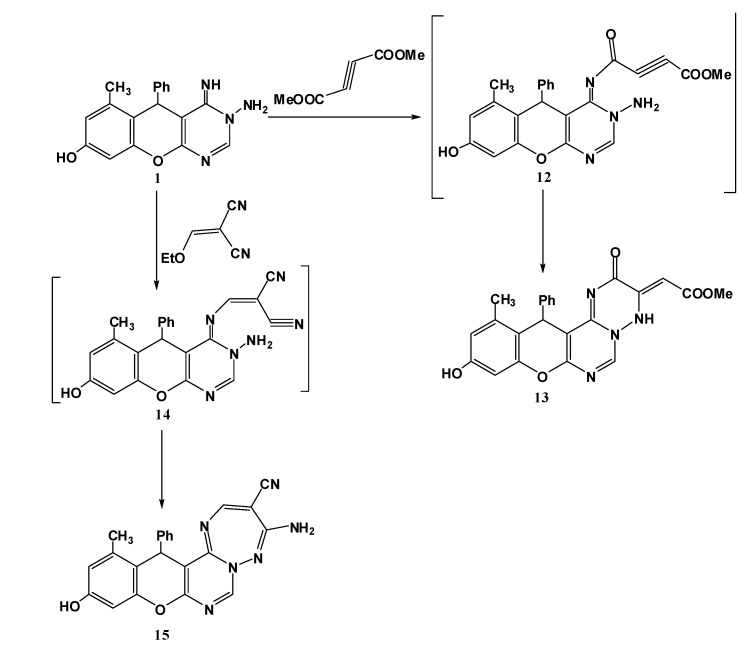
Reaction of title compound **1** with activated unsaturated compounds.

Consequently, we aimed to investigate further the behaviour of the aminopyrimidine **1** towards activated unsaturated compounds such as dimethyl acetylenedicarboxylate and ethoxymethylene malonitrile. The reactions were performed without catalyst in ethanol. It was found that this reaction proceeds in a simple manner through addition to acetylenic function or the olefinic double bond followed by loss of methanol or ethanol to furnish the expected triazine derivative **13** or the triazepine derivative **15** respectively (Scheme 4).

Confirmatory evidence for the structure assignment for compound **13** was provided by spectroscopic data. The IR spectrum revealed absorption bands at 1665, 1712 cm^−1^ characteristic for C=O, and COOMe; in the ^1^H-NMR spectrum, two signals at 2.17, 3.48 ppm assignable to two CH_3_ groups (CH_3_, OCH_3_) a more characteristic signal at 5.60 ppm integrating for one proton (=CHCOOMe).

The spectroscopic data for compound **15** were in a good agreement with this proposed structure, IR should show no great difference, while the electron ionization mass spectrum was consistent with the expected molecular mass for the proposed structure (*m/z* = 326). Furthermore, the ^1^H-NMR spectrum displayed a new signal at 8.57 ppm attributable to H5 in the trizepine ring.

**Scheme 5:**
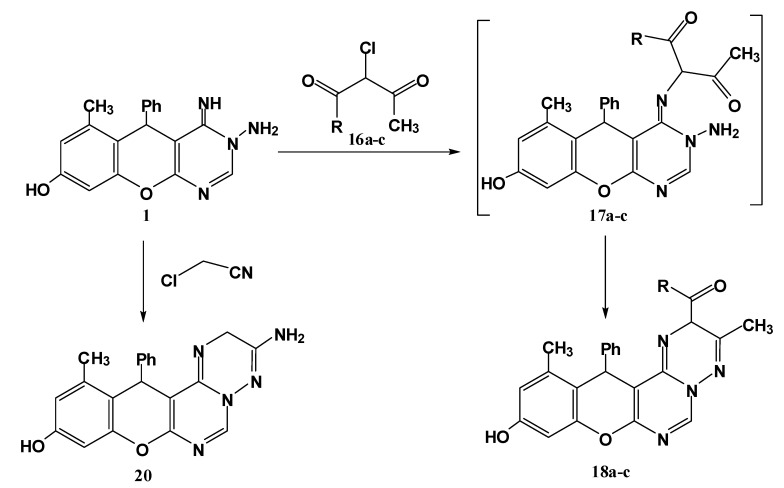
Synthesis of triazine derivatives **18a**–**c**and **20**.

Finally, an additional pathway for synthesis of substituted triazine derivatives **18a**–**c** was achieved through reaction of the title compound **1** with α-halo compounds (namely ethyl α-chloroacetoacetate, α-chloroacetylacetone and α-chloroacetoacetanilide) in refluxing ethanol containing triethylamine (Scheme 5).

The reaction proceeds through nucleophilic substitution followed by cyclocondensation. The structural assignment of these compounds was based on spectral evidence and microanalyses. The mass spectra of these products **18a**–**c** showed the molecular ion peaks at the expected *m/z* values. In their IR spectra, the appearance of absorption bands in the range 1712–1660 cm^−1^ confirmed the presence of a C=O group. The ^1^H-NMR spectrum, for example for compound **18a**, revealed two signals at 2.21, 2.23 ppm each integrating for three protons (CH_3_-phenolic ring, CH_3_-triazine ring) in addition to the characteristic ethoxy triplet-quartet pattern; a new characteristic signal at 5.50 ppm assignable for H5 in triazine ring. In a similar manner, alkylation of the imino function of compound **1** with chloroacetonitrile followed by *in situ* cyclization through the addition of the amino group to the cyano function delivered the aminotriazine derivative **20** (Scheme 5).

### 2.2. Antitumor Screening Test

**Figure 1:**
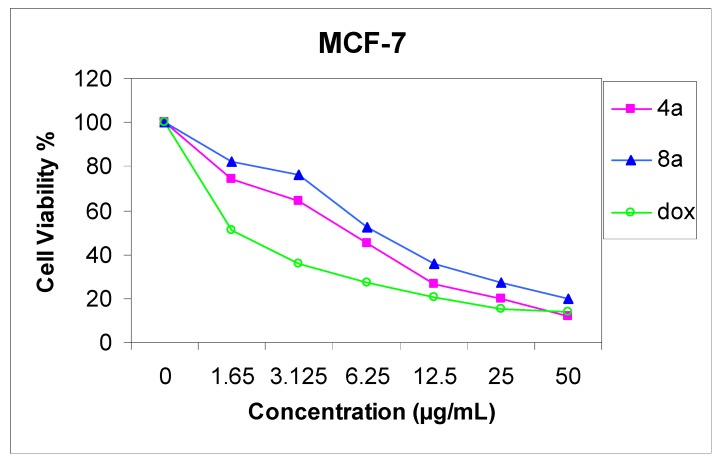
Effect of concentration of compound **4a** and **8a** on MCF-7 cell.

**Figure 2:**
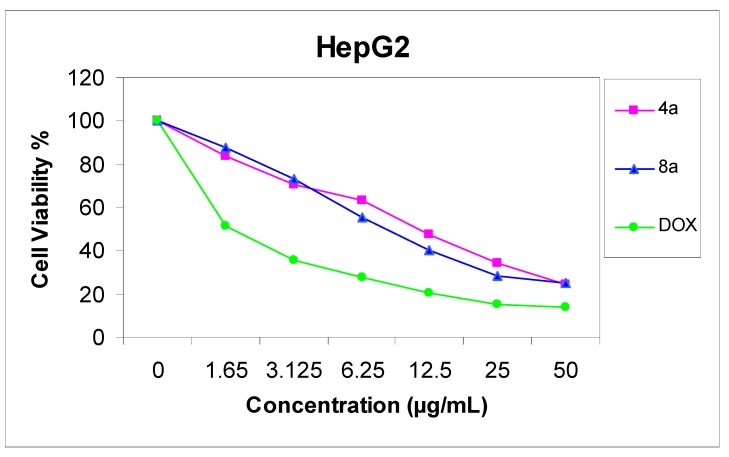
Effect of concentration of compound **4a** and **8a** on HepG2 cell.

The cytotoxicity of compounds **4a** and **8a** was evaluated against two cell lines representing two common forms of human cancer i.e. human hepatocellular carcinoma cell line (HepG2) and human breast adenocarcinoma cell line (MCF-7). For comparison purposes, the cytotoxicity of doxorubicin, a standard antitumor drug, was evaluated under the same conditions (IC_50_ value of doxorubicin = 0.59 ± 0.04 and 0.72 ± 0.08 µg/mL, respectively). The analysis of the data obtained indicated that the IC_50_ values (dose of the compound which causes a 50% reduction of survival values) for such compounds against human breast cell MCF-7 line are 5.36 ± 0.12 and 6.71 ± 0.09 µg/mL, respectively (Figure 1), but against liver carcinoma cell line HepG2 they are 9.94 ± 0.15 and 6.93 ± 0.08 µg/mL, respectively (Figure 2). All values were calculated from dose-response curve done in triplicate for each compound. Values were given ± standard deviation. The value of IC_50_ indicated that:

(1) Generally, both the tested compounds tended to be more active cytotoxic agents against human breast cell MCF-7 line, than HepG2 cell line;
(2) Compound **4a** is a more active cytotoxic agent against human breast cell MCF-7 line;
(3) Compound **8a** is a more active cytotoxic agent against human hepatocellular carcinoma cell line HepG2.

## 3. Experimental

### 3.1. Chemistry

#### 3.1.1. General

Melting points were determined on a Gallenkamp apparatus and are uncorrected. IR spectra were recorded in a Pye-Unicam SP300 instrument in potassium bromide discs. ^1^H-NMR spectra were recorded in a Varian Mercury VXR-300 spectrometer at 300 MHz in DMSO-*d*_6_ and the chemical shifts were related to TMS as standard solvent. Mass spectra were recorded in a GCMS-QP 1000 EX Shimadzu spectrometer, the ionizing voltage was 70 eV. Elemental analyses were carried out at the Microanalytical Laboratory of Cairo University, Giza, Egypt. Antitumor activity was evaluated by the Regional Center for Mycology and Biotechnology, Al-Azhar University, Cairo, Egypt. 3-Amino-8-hydroxy-4-imino-6-methyl-5-phenyl-4,5-dihydro-3*H*-chromeno[2,3-d]pyrimidine (**1**) [10] and hydrazonoyl halides **2** [26,27] were prepared as reported in the literature.

#### 3.1.2. Synthesis of 10-Hydroxy-3-substituted-12-methyl-13-phenyl-2-(2-substituted phenyl-hydrazono)-2*H*,13*H*-chromeno[2,3-d]pyrimido[1,6-b][1,2,4]triazines **4a**–**l**

*General procedure*: A mixture of 3-amino-8-hydroxy-4-imino-6-methyl-5-phenyl-4,5-dihydro-3*H*-chromeno[2,3-d] pyrimidine (**1**, 0.32 g, 1 mmol) and the appropriate hydrazonoyl halide **2** (1 mmol) in ethanol (20 mL) was refluxed for 2 h (monitored by TLC), then allowed to cool and the solid formed was filtered off, washed with ethanol, dried and recrystallized from DMF to give **4a**–**l**.

*10-Hydroxy-3,12-dimethyl-13-phenyl-2-(2-phenylhydrazono)-2H,13H-chromeno[2,3-d]pyrimido[1,6-b][1,2,4]triazine *(**4a**). Yield 71%; reddish-brown solid; mp. 334 °C; IR (KBr): *v* 1630 (C=N), 3427 (br, OH, NH) cm^−1^; ^1^H-NMR (DMSO-*d*_6_): δ_H_ 2.14 (3H, s, 12-CH_3_), 2.16 (3H, s, 3-CH_3_), 5.40 (1H, s, 13-H), 6.45 (1H, s, 9-H), 6.52 (1H, s, 11-H), 6.75–7.45 (10H, m, Ar-H), 8.39 (1H, s, 6-H), 9.30 (1H, br s, NH), 9.66 (1H, s, OH); MS *m/z* (%): 463 (M^+^ + 1, 38), 462 (M^+^, 100), 385 (23), 192 (24), 77 (33). Anal. Calcd for C_27_H_22_N_6_O_2_ (462.18): C, 70.12; H, 4.79; N, 18.17. Found C, 70.10; H, 4.65; N, 18.03%.

*10-Hydroxy-3,12-dimethyl-13-phenyl-2-[2-(p-tolyl)hydrazono]-2H,13H-chromeno[2,3-d]-pyrimido[1,6-b][1,2,4] triazine *(**4b**). Yield 74%; reddish-brown solid; mp. 345 °C; IR (KBr): *v* 1639 (C=N), 3421 (br, OH,NH) cm^−1^; ^1^H-NMR (DMSO-*d*_6_): δ_H_ 2.18 (3H, s, 12-CH_3_), 2.19 (3H, s, p-toly-CH_3_), 2.20 (3H, s, 3-CH_3_), 5.44 (1H, s, 13-H), 6.48 (1H, s, 9-H), 6.56 (1H, s, 11-H), 6.82–7.55 (9H, m, Ar-H), 8.42 (1H, s, 6-H), 9.37 (1H, br s, NH), 9.72 (1H, s, OH); MS *m/z* (%): 477 (M^+^ + 1, 34), 476 (M^+^, 100), 399 (14), 253 (18), 200 (22), 77 (28). Anal. Calcd for C_28_H_24_N_6_O_2_ (476.20): C, 70.57; H, 5.08; N, 17.64. Found C, 70.51; H, 5.11; N, 17.34%.

*2-[2-(4-Chlorophenyl)hydrazono]-10-Hydroxy-3,12-dimethyl-13-phenyl-2H,13H-chromeno[2,3-d]pyrimido[1,6-b][1,2,4]triazine* (**4c**). Yield 76%; reddish-brown solid; mp. 352 °C; IR (KBr): *v* 1639 (C=N), 3448 (br, OH,NH) cm^−1^; ^1^H-NMR (DMSO-*d*_6_): δ_H_ 2.19 (3H, s, 12-CH_3_), 2.21 (3H, s, 3-CH_3_), 5.42 (1H, s, 13-H), 6.49 (1H, s, 9-H), 6.58 (1H, s, 11-H), 6.88–7.63 (9H, m, Ar-H), 8.40 (1H, s, 6-H), 9.39 (1H, br s, NH), 9.75 (1H, s, OH); MS *m/z* (%): 498 (M^+^ + 2, 36), 497 (M^+^ + 1, 23), 496 (M^+^, 100), 428 (27), 253 (18), 209 (46), 77 (60), 55 (73). Anal. Calcd for C_27_H_21_ClN_6_O_2_ (496.14): C, 65.26; H, 4.26; N, 16.91. Found C, 65.10; H, 4.34; N, 16.69%.

*10-Hydroxy-3,12-dimethyl-2-[2-(4-nitrophenyl)hydrazono]-13-phenyl--2H,13H-chromeno[2,3-d]pyrimido[1,6-b][1,2,4]triazine* (**4d**). Yield 76%; dark red solid; mp. 358 °C; IR (KBr): *v* 1639 (C=N), 3440 (br, OH,NH) cm^−1^; ^1^H-NMR (DMSO-*d*_6_): δ_H_ 2.20 (3H, s, 12-CH_3_), 2.21 (3H, s, 3-CH_3_), 5.48 (1H, s, 13-H), 6.48 (1H, s, 9-H), 6.60 (1H, s, 11-H), 6.89–7.77 (9H, m, Ar-H), 8.43 (1H, s, 6-H), 9.42 (1H, br s, NH), 9.77 (1H, s, OH); MS *m/z* (%): 508 (M^+^ + 1, 17), 507 (M^+^, 46), 429 (20), 294 (37), 253 (50), 77 (44), 55 (100). Anal. Calcd for C_27_H_21_N_7_O_4_ (507.17): C, 63.90; H, 4.17; N, 19.32. Found C, 63.76; H, 4.02; N, 19.12%.

*2-[2-(4-Bromophenyl)hydrazono]-10-Hydroxy-3,12-dimethyl-13-phenyl-2H,13H-chromeno[2,3-d]pyrimido[1,6-b][1,2,4]triazine* (**4e**). Yield 71%; dark reddish-brown solid; mp. 358 °C; IR (KBr): *v* 1633 (C=N), 3452 (br, OH, NH) cm^−1^; ^1^H-NMR (DMSO-*d*_6_): δ_H_ 2.18 (3H, s, 12-CH_3_), 2.20 (3H, s, 3-CH_3_), 5.43 (1H, s, 13-H), 6.51 (1H, s, 9-H), 6.60 (1H, s, 11-H), 6.90–7.69 (9H, m, Ar-H), 8.41 (1H, s, 6-H), 9.38 (1H, br s, NH), 9.69 (1H, s, OH); MS *m/z* (%): 542 (M^+^ + 2, 93), 541 (M^+^ + 1, 71), 540 (M^+^, 100),463 (13), 253 (25), 90 (40), 77 (9). Anal. Calcd for C_27_H_21_BrN_6_O_2_ (540.09): C, 59.90; H, 3.91; N, 15.52. Found C, 59.70; H, 3.86; N, 15.37.%.

*10-Hydroxy-2-[2-(4-methoxyphenyl)hydrazono]-3,12-dimethyl-13-phenyl-2H,13H-chromeno[2,3-d]pyrimido[1,6-b][1,2,4]triazine* (**4f**). Yield 74%; dark red solid; mp. 320 °C; IR (KBr): *v* 1638 (C=N), 3412 (br, OH and NH) cm^−1^; ^1^H-NMR (DMSO-*d*_6_): δ_H_ 2.12 (3H, s, 12-CH_3_), 2.15 (3H, s, 3-CH_3_), 3.71 (3H, s, OCH_3_), 5.37 (1H, s, 13-H), 6.45 (1H, s, 9-H), 6.51 (1H, s, 11-H), 6.86–7.44 (9H, m, Ar-H), 8.35 (1H, s, 6-H), 9.14 (1H, br s, NH), 9.65 (1H, s, OH); MS *m/z* (%): 493 (M^+^ + 1, 33), 492 (M^+^, 100), 415 (10), 253 (15), 122 (31), 77 (22). Anal. Calcd for C_28_H_24_N_6_O_3_ (492.19): C, 68.28; H, 4.91; N, 17.06. Found C, 68.11; H, 4.78; N, 16.89%.

*10-Hydroxy-12-methyl-3,13-diphenyl-2-(2-phenylhydrazono)-2H,13H-chromeno[2,3-d]pyrimido[1,6-b][1,2,4]triazine* (**4g**). Yield 70%; orange solid; mp. 316 °C; IR (KBr): *v* 1637 (C=N), 3438 (br, OH,NH) cm^−1^; ^1^H-NMR (DMSO-*d*_6_): δ_H_ 2.19 (3H, s, 12-CH_3_), 5.47 (1H, s, 13-H), 6.51 (1H, s, 9-H), 6.59 (1H, s, 11-H), 6.73–7.85 (15H, m, Ar-H), 8.47 (1H, s, 6-H), 9.48 (1H, br s, NH), 9.74 (1H, s, OH); MS *m/z* (%): 524 (M^+^, 16), 384 (12), 228 (100), 77 (20). Anal. Calcd for C_32_H_24_N_6_O_2_(524.20): C, 73.27; H, 4.61; N, 16.02. Found C, 73.12; H, 4.45; N, 15.22%.

*10-Hydroxy-12-methyl-3,13-diphenyl-2-[2(p-tolyl)hydrazono]-2H,13H-chromeno[2,3-d]pyrimido[1,6-b][1,2,4]triazine* (**4h**). Yield 74%; dark brown solid; mp. 312 °C; IR (KBr): *v* 1628 (C=N), 3427 (br, OH and NH) cm^−1^; ^1^H-NMR (DMSO-*d*_6_): δ_H_ 2.18 (3H, s, 12-CH_3_), 2.50 (3H, s, *p*-tolyl-CH_3_), 5.44 (1H, s, 13-H), 6.47 (1H, s, 9-H), 6.54 (1H, s, 11-H), 6.76–7.87 (14H, m, Ar-H), 8.42 (1H, s, 6-H), 9.44 (1H, br s, NH), 9.64 (1H, s, OH); MS *m/z* (%): 540 (M^+^ + 2, 9), 539 (M^+^ + 1, 42), 538 (M^+^, 100), 316 (10), 238 (17), 77 (27). Anal. Calcd for C_33_H_26_N_6_O_2_ (538.21): C, 73.59; H, 4.87; N, 15.60. Found C, 73.59; H, 4.87; N, 15.60%.

*2-[2-(4-Chlorophenyl)hydrazono]-10-Hydroxy-12-methyl-3,13-diphenyl2H,13H-chromeno-[2,3-d]pyrimido[1,6-b][1,2,4]triazine* (**4i**). Yield 72%; dark brown solid; mp. 330 °C; IR (KBr): *v* 1643 (C=N), 3444 (br, OH and NH) cm^−1^; ^1^H-NMR (DMSO-*d*_6_): δ_H_ 2.15 (3H, s, 12-CH_3_), 5.43 (1H, s, 13-H), 6.40 (1H, s, 9-H), 6.51 (1H, s, 11-H), 6.72–7.98 (14H, m, Ar-H), 8.43 (1H, s, 6-H), 9.45 (1H, br s, NH), 9.70 (1H, s, OH); MS *m/z* (%): 559 (M^+^ + 1, 9), 558 (M^+^, 21), 391 (12), 201 (21), 105 (47), 77 (47), 55 (100). Anal. Calcd for C_32_H_23_ClN_6_O_2_ (558.16): C, 68.75; H, 4.15; N, 15.03. Found C, 68.73; H, 4.10; N, 15.00%.

*2-[2-(3-Bromophenyl)hydrazono]-10-Hydroxy-12-methyl-3,13-diphenyl-2H,13H-chromeno-[2,3-d]pyrimido[1,6-b][1,2,4] triazine* (**4j**). Yield 70%; dark brown solid; mp. 180 °C; IR (KBr): *v* 1643 (C=N), 3413 (br, OH and NH) cm^−1^; ^1^H-NMR (DMSO-*d*_6_): δ_H_ 2.17 (3H, s, 12-CH_3_), 5.46 (1H, s, 13-H), 6.48 (1H, s, 9-H), 6.56 (1H, s, 11-H), 6.69–7.90 (14H, m, Ar-H), 8.47 (1H, s, 6-H), 9.50 (1H, br s, NH), 9.74 (1H, s, OH); MS *m/z* (%): 604 (M^+^ + 2, 3), 603 (M^+^ + 1, 4), 602 (M^+^, 3), 503 (4), 471 (5), 305 (15), 228 (88), 105 (100), 77 (79). Anal. Calcd for C_32_H_23_BrN_6_O_2_ (602.11): C, 63.69; H, 3.84; N, 13.93. Found C, 63.57; H, 3.75; N, 13.68%.

*Ethyl4-[10-hydroxy-12-methyl-3,13-diphenyl-2H,13H-chromeno[2,3-d]pyrimido[1,6-b][1,2,4]triazin-2-ylidene)hydrazinyl]benzoate* (**4k**). Yield 72%; dark brown solid; mp. 352 °C; IR (KBr): *v* 1639 (C=N), 1715 (C=O), 3420 (br, OH and NH) cm^−1^; ^1^H-NMR (DMSO-*d*_6_): δ_H_ 1.34 (3H, t, CH_3_), 2.21 (3H, s, 12-CH_3_), 4.51 (2H, q, CH_2_), 5.51 (1H, s, 13-H), 6.52 (1H, s, 9-H), 6.58 (1H, s, 11-H), 6.70–7.92 (14H, m, Ar-H), 8.40 (1H, s, 6-H), 9.51 (1H, br s, NH), 9.70 (1H, s, OH); MS *m/z* (%): 597 (M^+^ + 1, 13), 596 (M^+^, 24), 567 (100), 524 (30), 432 (18), 228 (49), 103 (78), 77 (66). Anal. Calcd for C_35_H_28_N_6_O_4_ (596.22**)**: C, 70.46; H, 4.73; N, 14.09. Found C, 70.34; H, 4.45; N, 13.97%.

*10-Hydroxy-12-methyl-13-phenyl-3-2-(2-phenylhydrazono)-(thiophen-2-yl)-2H,13H-chromeno2,3-d]pyrimido[1,6-b][1,2,4]triazine* (**4l**). Yield 76%; dark red solid; mp. 228 °C; IR (KBr): *v* 1647 (C=N), 3387 (br, OH,NH) cm^−1^; ^1^H-NMR (DMSO-*d*_6_): δ_H_ 2.19 (3H, s, 12-CH_3_), 5.46 (1H, s, 13-H), 6.48 (1H, s, 9-H), 6.56 (1H, s, 11-H), 6.68–7.96 (13H, m, Ar-H), 8.49 (1H, s, 6-H), 9.54 (1H, br s, NH), 9.71 (1H, s, OH); MS *m/z* (%): 531 (M^+^ + 1, 2), 530 (M^+^ , 7), 385 (9), 306 (39), 228 (100), 77 (29). Anal. Calcd for C_30_H_22_N_6_O_2_S (530.15): C, 67.91; H, 4.18; N, 15.84. Found C, 67.91; H, 4.18; N, 15.84%.

#### 3.1.3. Synthesis of 10-hydroxy-12-methyl-9-phenylazo-2,13-diphenyl-13*H*-chromeno[2,3-d]pyrimido [1,6-b][1,2,4] triazine **6**

*Synthesis of 10-hydroxy-12-methyl-2,13-diphenyl-13H-chromeno[2,3-d]pyrimido[1,6-b][1,2,4]triazine* (**5**). A mixture of **1** (0.320 g, 1 mmol) and phenacyl bromide (0.198 g, 1 mmol) in absolute ethanol (30 mL) was refluxed for 2 h (monitored by TLC). The product started to separate out during the course of reaction. The crystalline solid was filtered, washed with water, dried and recrystallized from dioxane to give compound **5** in 76% yield as yellow solid; mp. 230 °C; IR (KBr): *v* 1632 (C=N), 3425 (OH) cm^−1^; ^1^H-NMR (DMSO-*d*_6_): δ_H_ 2.14 (3H, s, 12-CH_3_), 4.23 (2H, s, CH_2_), 5.55 (1H, s, 13-H), 6.52 (1H, s, 9-H), 6.54 (1H, s, 11-H), 7.18–7.38 (10H, m, Ar-H),), 8.63 (1H, s, 6-H), 9.82 (1H, s, OH); MS *m/z* (%): 421 (M^+^ +1, 1), 420 (M^+^, 2), 329 (14), 305 (16), 253 (21), 228 (100), 201 (23), 105 (15), 77 (26); Anal. Calcd for C_26_H_20_N_4_O_2_ (420.47): C, 74.27; H, 4.79; N, 13.32. Found C, 74.14; H, 4.87; N, 13.35%.

*Coupling of ***5*** with benzenediazonium chloride*. To a solution of **5** (0.421g, 1 mmol) in ethanol (20 mL) was added sodium acetate trihydrate (0.138 g, 1 mmol), and the mixture was cooled to 0–5 °Cin an ice bath. To the resulting cold solution was added portionwise a cold solution of benzenediazonium chloride (1 mmol) [prepared by diazotizing aniline] dissolved in hydrochloric acid (6 M, 1 mL) with a solution of sodium nitrite (0.07 g, 1 mmol) in water (2 mL). After complete addition of the diazonium salt, the reaction mixture was stirred for a further 30 min in an ice bath. The solid that separated was filtered off, washed with water and finally recrystallized from ethanol to give product **6**. Yield 78%; orange solid; mp. 298 °C; IR (KBr): *v* 1634 (C=N), 3466 (br, OH and NH) cm^−1^; ^1^H-NMR (DMSO-*d*_6_): δ_H_ 2.16 (3H, s, 12-CH_3_), 4.24 (2H, s, CH_2_), 5.56 (1H, s, 13-H), 6.54 (1H, s, 11-H), 7.03–7.41 (15H, m, Ar-H), 8.63 (1H, s, 6-H), 9.88 (1H, s, OH); MS *m/z* (%): 524 (M^+^, 23), 305 (100), 228 (87), 77 (64). Anal. Calcd for C_32_H_24_N_6_O_2_(524.20): C, 73.27; H, 4.61; N, 16.02. Found C, 73.10; H, 4.65; N, 15.12%.

#### 3.1.4. Synthesis of 4-substituted-11-hydroxy-13-methyl-14-phenyl-3*H*,14*H*-chromeno[2,3-d]pyrimido [1,6-b][1,2,4] triazepin-2(3*H*)-ones **8a,b**.

A mixture of compound **1** (0.32 g, 1 mmol) and ethyl acetoacetate or ethyl benzoylacetate (1.5 mmol) was heated under reflux for 2 h. After cooling, the solid precipitated was collected and crystallized from dioxane to give **8a,b**, respectively.

*11-Hydroxy-4,13-dimethyl-14-phenyl-3H,14H-chromeno[2,3-d]pyrimido[1,6-b][1,2,4]triazepin-2(3H)-one* (**8a**). Yield 74%; yellow solid; mp. 189 °C; IR (KBr): *v* 1635 (C=N), 1720 (CO), 3251 (NH), 3406 (OH) cm^−1^; ^1^H-NMR (DMSO-*d*_6_): δ_H_ 2.07 (3H, s, 13-CH_3_), 2.17 (3H, s, 4-CH_3_), 4.06 (2H, s, CH_2_), 5.57 (1H, s, 14-H), 6.50 (1H, s, 10-H), 6.60 (1H, s, 12-H), 7.14–7.29 (5H, m, Ar-H), 9.50 (1H, s, 7-H), 9.72 (1H, s, OH); MS *m/z* (%): 388 (M^+^ + 2, 1), 387 (M^+^ + 1, 5), 386 (M^+^, 16), 309 (100), 266 (19) 77 (11), 55. Anal. Calcd for C_22_H_18_N_4_O_3_ (386.14): C, 68.38; H, 4.70; N, 14.50. Found C, 68.32; H, 4.65; N, 14.36%.

*11-Hydroxy-13-methyl-4,14-diphenyl-3H,14H-chromeno[2,3-d]pyrimido[1,6-b][1,2,4]triazepin-2(3H)-one* (**8b**). Yield 74%; yellow solid; mp. 214 °C; IR (KBr): *v* 1632 (C=N), 1702 (CO), 3416 (OH) cm^−1^; ^1^H-NMR (DMSO-*d*_6_): δ_H_ 2.10 (3H, s, 13-CH_3_), 4.15 (2H, s, CH_2_), 5.62 (1H, s, 14-H), 6.55 (1H, s, 10-H), 6.68 (1H, s, 12-H), 7.06–7.67 (10H, m, Ar-H), 9.52 (1H, s, 7-H), 9.82 (1H, s, OH); MS *m/z* (%): 449 (M^+^+1, 8), 448 (M^+^, 20), 371 (100), 266 (24), 105 (76), 77 (75). Anal. Calcd for C_27_H_20_N_4_O_3_ (448.15): C, 72.31; H, 4.49; N, 12.49. Found C, 72.18; H, 4.34; N, 12.29%.

#### 3.1.5. Reaction of *N*-aminopyrimidine **1** with Aromatic Aldehydes

*General procedure:* An appropriate aromatic aldehyde (1 mmol.) was added to a solution of the *N*-aminopyrimidine **1** (0.32 g, 1 mmol) in absolute ethanol (15 mL) containing a few drops of piperidine, the resulting mixture refluxed for 3 h. The solids formed after cooling were collected by filtration, washed with ether and crystallized from DMF.

*9-Hydroxy-11-methyl-12-phenyl-2-(4-chlorophenyl)-12H-chromeno[3,2-e][1,2,4]triazolo[1,5-c]pyrimidine* (**10a**). Yield 76%; yellow solid; mp. 270 °C; IR (KBr): *v* 1639 (C=N), 3441 (br, OH) cm^−1^; ^1^H-NMR (DMSO-*d*_6_): δ_H_ 2.20 (3H, s, 11-CH_3_), 6.01 (1H, s, 12-CH), 5.86 (1H, s, NH), 6.47 (1H, s, 8-H), 6.55 (1H, s, 10-H), 7.08–8.40 (9H, m, Ar-H), 9.67 (1H, s, 5-H), 11.36 (1H, s, OH); MS *m/z* (%): 440 (M^+^, 2), 366 (12), 305 (100), 228 (69), 201 (33), 138(73), 77(52). Anal.Calcd for C_25_H_17_ClN_4_O_2_S (440.10): C, 68.11; H, 3.89; N, 12.71. Found C, 68.01; H, 3.67; N, 12.56%.

*2-(2-Chlorophenyl)-9-hydroxy-11-methyl-12-phenyl-12H-chromeno[3,2-e][1,2,4]triazolo[1,5-c]pyrimidine* (**10b**). Yield 74%; yellow solid; mp. 252 °C; IR (KBr): *v* 1636 (C=N), 3438 (OH) cm^−1^; ^1^H-NMR (DMSO-*d*_6_): δ_H_ 2.21 (3H, s, 11-CH_3_), 6.02 (1H, s, 12-H), 6.48 (1H, s, 8-H), 6.52 (1H, s, 10-H), 7.05–8.33 (9H, m, Ar-H), 9.60 (1H, s, 5-H), 11.03 (1H, s, OH); MS *m/z* (%): 440 (M^+^, 12), 305 (66), 228 (100), 138(58), 77(47). Anal.Calcd for C_25_H_17_ClN_4_O_2_S (440.10): C, 68.11; H, 3.89; N, 12.71. Found C, 68.21; H, 3.71; N, 12.49%.

*2-(2,4-Dichlorophenyl)-9-Hydroxy-11-methyl-12-phenyl-12H-chromeno[3,2-e][1,2,4]triazolo[1,5-c]pyrimidine* (**10c**). Yield 74%; yellow solid; mp. 298 °C; IR (KBr): *v* 1639 (C=N), 3444 (OH) cm^−1^; ^1^H-NMR (DMSO-*d*_6_): δ_H_ 2.25 (3H, s, 11-CH_3_), 6.12 (1H, s, 12-H), 5.88 (1H, s, NH), 6.49 (1H, s, 8-H), 6.61 (1H, s, 10-H), 7.08–8.46 (8H, m, Ar-H), 9.74 (1H, s, 5-H), 11.36 (1H, s, OH); MS *m/z* (%): 476 (M^+^ + 2, 7), 474 (M^+^, 6), 397 (26), 305 (24), 228 (75), 145 (100), 77 (38). Anal. Calcd for C_25_H_16_Cl_2_N_4_O_2_ (474.07): C, 63.17; H, 3.39; N, 11.79. Found C, 63.10; H, 3.21; N, 11.53%.

*9-Hydroxy-11-methyl-2-(3-nitrophenyl)-12-phenyl-12H-chromeno[3,2-e][1,2,4]triazolo[1,5-c]pyrimidine* (**10d**). Yield 72%; yellow solid; mp. 240 °C; IR (KBr): *v* 1632 (C=N), 3412 (OH) cm^−1^; ^1^H-NMR (DMSO-*d*_6_): 2.09 (3H, s, CH_3_), 5.58 (1H, s, 11-H), 6.42 (1H, s, 8- H), 6.57 (1H, s, 10-H), 7.10–8.46 (9H, m, Ar-H), 9.50 (1H, s, 5-H), 9.72 (1H, br s, OH); MS *m/z* (%): 452 (M^+^ + 1, 6), 451 (M^+^, 17), 374 (100), 172 (13),77 (18). Anal.Calcd for C_25_H_17_N_5_O_4_ (451.13): C, 66.51; H, 3.80; N, 15.51. Found C, 66.34; H, 3.65; N, 15.41%.

*3-(2-Hydroxybenzylideneamino)-4-imino-6-methyl-5-phenyl-4,5-dihydro-3H-chromeno[2,3-d]pyrimidin-8-ol* (**11**). Yield 78%; cannary yellow solid; mp. 192 °C; IR (KBr): *v* 1616 (C=N), 3433 (very br, 2OH,NH) cm^−1^; ^1^H-NMR (DMSO-*d*_6_): ^1^H-NMR δ_H_ 2.19 (3H, s, 6-CH_3_), 5.68 (1H, s, 5-H), 6.45 (1H, s, 9-H), 6.55 (1H, s, 7-H), 6.89–7.56 (9H, m, Ar-H), 8.27 (1H, s, CH=N-N), 8.37 (1H, s, 2-H), 8.53 (1H, s, NH), 8.57 (1H, s, OH); MS *m/z* (%):424 (M+, 29), 304 (100), 228 (94), 173 (35), 105 (29), 77 (75); Anal.Calcd for C_25_H_20_N_4_O_3_ (424.15): C, 70.74; H, 4.75; N, 13.20. Found C, 70.31; H, 4.56; N, 13.02%.

#### 3.1.6. Reaction of **1** with Activated Unsaturated Compounds

*Synthesis of 10-hydroxy-12-methyl-2-oxo-13-phenyl-2H,13H-chromeno[2,3-d]pyrimido[1,6-b][1,2,4]triazin-3(4H)-ylidene)acetate* (**13**). An equimolar mixture of **1** (0.32 g, 1 mmol) and dimethylacetylene dicarboxylate (0.142 g, 1 mmol) in methanol (20 mL) was refluxed for 2 h (monitored by TLC). The formed solid was collected by filtration and recrystallized from DMF to give compound **13**. Yield 78%; canary yellow solid; mp. 184 °C; IR (KBr): *v* = 1633 (C=N), 1665, 1712 (2C=O), 3466 (br, OH and NH) cm^−1^; ^1^H-NMR (DMSO-*d*_6_): δ_H_ 2.17 (3H, s, 12-CH_3_), 3.48 (3H, s, OCH_3_), 5.42 (1H, s, 13-H), 6.60 (1H, s, CH=CO_2_Me), 6.52 (1H, s, 9-H), 6.62 (1H, s, 11-H), 7.11–7.34 (5H, m, Ar-H), 9.12 (1H, s, 6-H), 9.95 (1H, s, NH), 10.15 (1H, s, OH); MS *m/z* (%): 432 (M^+^ + 2, 2), 431 (M^+^ + 1, 2), 430 (M^+^, 5), 305 (22), 253 (25), 228 (100), 105 (69), 77 (62). Anal. Calcd for C_23_H_18_N_4_O_5_ (430.13): C, 64.18; H, 4.22; N, 13.02. Found C, 64.12; H, 4.13; N, 12.82%.

*Synthesis of 4-Amino-11-hydroxy-13-methyl-14-phenyl-3H,14H-chromeno[2,3-d]pyrimido[1,6-b]1,2,4]triazepine-3-carbonitrile *(**15**). A mixture of **1** (0.32 g, 1 mmol) and ethoxymethylene malononitrile (0.122 g, 1 mmol) in methanol (20 mL) was refluxed for 1 h (monitored by TLC). The reaction mixture was cooled and the resulting precipitate was filtered off and recrystallized from DMF/EtOH to give **15**. Yield 76%; yellow solid; mp. 330 °C; IR (KBr): *v* 1628 (C=N), 2197 (CN), 3214, 3180 (NH2), 3466 (OH) cm^−1^; ^1^H-NMR (DMSO-*d*_6_): δ_H_ 2.07 (3H, s, 13-CH_3_), 5.61 (1H, s, 14-H), 6.50 (1H, s, 10-H), 6.60 (1H, s, 12-H), 7.12–7.33 (7H, m, Ar-H + NH_2_), 8.57 (1H, s, 2-H), 9.57 (1H, s, 7-H), 9.72 (1H, s, OH); MS *m/z* (%): 396 (M^+^, 3), 330 (13), 253 (100), 77 (11). Anal. Calcd for C_22_H_16_N_6_O_2_ (396.13): C, 66.66; H, 4.07; N, 21.20. Found C, 66.38; H, 4.01; N, 21.03%.

#### 3.1.7. Reaction of 1 with Active Chloromethylene Compounds **16a**–**c** and Chloroacetonitrile

*General procedure*: To a solution of **1** (0.32 g, 1 mmol) in ethanol was added triethylamine (0.7 mL) and the mixture was stirred for 10 min at room temperature. To the resulting clear solution was added active chloromethylene compounds **16a**–**c** and chloroacetonitrile (1 mmol) dropwise while stirring the reaction mixture. After complete addition, the reaction mixture was refluxed for 2 h (monitored by TLC). The solid that precipitated was filtered off, washed with H_2_O, dried and finally crystallized from ethanol to give the respective **18a**–**c** and **20**.

*2-Acetyl-10-hydroxy-3,12-dimethyl-13-phenyl-2H,13H-chromeno[2,3-d]pyrimido[1,6-b][1,2,4]triazine* (**18a**). Yield 74%; yellow solid; mp. 212 °C; IR (KBr): *v* 1639 (C=N), 1698 (C=O), 3410 (OH) cm^−1^; ^1^H-NMR (DMSO-*d*_6_): δ_H_ 2.17 (3H, s, 12-CH_3_), 2.19 (3H, s, 3-CH_3_), 2.25 (3H, s, CH_3_CO), 5.48 (1H, s, 2-H), 5.6 (1H, s, 13-H), 6.46 (1H, s, 9-H), 6.58 (1H, s, 11-H), 7.14–7.54 (5H, m, Ar-H), 8.20 (1H, s, 6-H), 9.69 (1H, br s, OH); MS *m/z* (%): 400 (M^+^, 8), 305 (12), 276 (100), 253 (11), 228 (92), 105 (18), 77 (15). Anal. Calcd for C_23_H_20_N_4_O_3_ (400.15): C, 68.99; H, 5.03; N, 13.99. Found C, 68.78; H, 4.83; N, 13.86%.

*Ethyl-10-hydroxy-3,12-dimethyl-13-phenyl-2H,13H-chromeno[2,3-d]pyrimido[1,6-b][1,2,4]triazine-2-carboxylate* (**18b**). Yield 74%; yellow solid; mp. 160 °C; IR (KBr): *v* 1634 (C=N), 1712 (C=O), 3422 (OH) cm^−1^; ^1^H-NMR (DMSO-*d*_6_): δ_H_ 1.30 (3H, t, CH_3_), 2.21 (3H, s, 12-CH_3_), 2.23 (3H, s, 3-CH_3_), 4.22 (2H, q, CH_2_), 5.50 (1H, s, 2-H), 5.58 (1H, s, 13-H), 6.40 (1H, s, 9-H), 6.49 (1H, s, 11-H), 7.11–7.36 (5H, m, Ar-H), 8.17 (1H, s, 6-H), 9.61 (1H, br s, OH); MS *m/z* (%): 430 (M^+^, 12), 354 (16), 305 (59), 268 (43), 228 (100), 105 (18), 76 (52). Anal. Calcd for C_24_H_22_N_4_O_4_ (430.16): C, 66.97; H, 5.15; N, 13.02. Found C, 66.76; H, 5.01; N, 12.92%.

*10-Hydroxy-3,12-dimethyl-N,13-diphenyl-2H,13H-chromeno[2,3-d]pyrimido[1,6-b][1,2,4] triazine-2-carboxamide* (**18c**). Yield 76%; yellow solid; mp. 198 °C; IR (KBr): *v* 1636 (C=N), 1660 (C=O), 3433 (br, OH and NH) cm^−1^. ^1^H-NMR (DMSO-*d*_6_): δ_H_ 2.14 (3H, s, 12-CH_3_), 2.17 (3H, s, 3-CH_3_), 5.37 (1H, s, 2-H), 5.59 (1H, s, 13-H), 6.40 (1H, s, 9-H), 6.52 (1H, s, 11-H), 7.19–7.84 (10H, m, Ar-H), 8.18 (1H, s, 6-H), 9.45 (1H, s, NH), 9.79 (1H, br s, OH); MS *m/z* (%): 477 (M^+^, 16), 305 (100), 268 (43), 228 (94), 105 (46), 77 (68). Anal. Calcd for C_28_H_23_N_5_O_3_ (477.18): C, 70.43; H, 4.85; N, 14.67. Found C, 70.33; H, 4.74; N, 14.62%.

*3-Amino-10-hydroxy-3,12-dimethyl-13-phenyl-2H,13H-chromeno[2,3-d]pyrimido[1,6-b][1,2,4]triazine* (**20**). Yield 73%; yellow solid; mp. 165 °C; IR (KBr): *v* 1639 (C=N), 3356, 3198 (NH_2_), 3406 (OH) cm^−1^. ^1^H-NMR (DMSO-*d*_6_): δ_H_ 2.11 (3H, s, 12-CH_3_), 3.88 (2H, s, CH_2_), 5.57 (1H, s, 13-H), 6.35 (2H, br s, NH_2_), 6.52 (1H, s, 9-H), 6.50 (1H, s, 11-H), 7.11–7.42 (5H, m, Ar-H), 8.67 (1H, s, 6-H), 9.84 (1H, s, OH); MS *m/z* (%): 359 (M^+^, 3), 253 (25), 228 (100), 77 (23). Anal. Calcd for C_20_H_17_N_5_O_2_ (359.14): C, 66.84; H, 4.77; N, 19.49. Found C, 66.67; H, 4.54; N, 19.36%.

### 3.2. Cytotoxic Activity

Potential cytotoxicity of the compounds was tested using the method of Skehan *et al*. [28], using Sulfo-Rhodamine-B stain (SRB). Cells were plated in 96-multiwill plates (10^4^ cells/well) for 24 h before treatment with the tested compound to allow attachment of cell to the wall of the plate. Different concentrations of the compound under test (0, 1.56, 3.125, 6.25, 12.5, 25, and 50 µg/mL) were added to the cell monolayer in triplicate wells individual dose, monolayer cells were incubated with the compounds for 48 h at 37 °C and in atmosphere of 5% CO_2_. After 48 h, cells were fixed, washed and stained with SRB stain, excess stain was washed with acetic acid and attached stain was recovered with *tris*-EDTA buffer, color intensity was measured in an ELISA reader. The relation between surviving fraction and drug concentration is plotted to get the survival curve of each tumor cell line after the specified compound and the IC_50_ was calculated (Figure 1 and Figure 2).

## 4. Conclusions

In this report, a simple method for the synthesis of new chromeno[2,3-d]]pyrimido[1,6-b][1,2,4]triazines, chromeno[2,3-d]]pyrimido[1,6-b][1,2,4] triazepinones and chromeno[3,2-e][1,2,4]triazolo[1,5-c]pyrimidines by the reactions of 3-amino-8-hydroxy-4-imino-6-methyl-5-phenyl-4,5-dihydro-3*H*-chromeno[2,3-d]pyrimidine and hydrazonoyl halides, ethyl acetoacetate, ethyl benzoylacetate, and aromatic aldehydes are demonstrated. The new compounds **4a** and **8a** have been evaluated for the antitumor activity against human breast cell MCF-7 line and liver carcinoma cell line HepG2.

## References

[1] Sabry N.M., Mohamed H.M., Khattab E.S., Motlaq S.S., El-Agrody A.M. (2011). Synthesis of 4*H*-chromene, coumarin, 12*H*-chromeno[2,3-d]pyrimidine derivatives and some of their antimicrobial and cytotoxicity activities. Eur. J. Med. Chem..

[2] Rai U.S., Isloor A.M., Shetty P., Vijesh A.M., Prabhu N., Isloor S., Thiageeswaran M., Fun H.K. (2010). Novel chromeno[2,3-*b*]-pyrimidine derivatives as potential anti-microbial agents. Eur. J. Med. Chem..

[3] Abd El-Wahab H.F., Mohamed A.M., El-Agrody H.M., El-Nassag A.A., Bedair M.H. (2012). Synthesis and Reactions of Some New Benzylphthalazin-1-ylaminophenols, 2*H*-Chromene and 5*H*-Chromeno[2,3-d]pyrimidine Derivatives with Promising Antimicrobial Activities. Lett. Org. Chem..

[4] Kamdar N.R., Haveliwala D.D., Mistry P.T., Patel S.K. (2011). Synthesis and evaluation of *in vitro* antitubercular activity and antimicrobial activity of some novel 4*H*-chromeno[2,3-*d*]pyrimidine via 2-amino-4-phenyl-4*H*-chromene-3-carbonitriles. Med. Chem. Res..

[5] Attaby F.A., Eldin S.M. (1999). Synthesis of Pyrimidine, Thiazolopyrimidine, Pyrimidotriazine and Triazolopyrimidine Derivatives and their Biological Evaluation. Z. Naturforsch..

[6] Aly A.A., Gad El-Karim I.A. (2011). Facile synthesis of new pyrazolopyrimidine derivatives of potential biosignificant interest. J. Korean Chem. Soc..

[7] El-Mahdy K.M., Abdel-Rahman R.M.A. (2011). Convenient methods for synthetic isomeric structures of pyrimido-1,2,4-triazine derivatives as biocidal agents. Acta Chim. Slov..

[8] Guertin K.R., Setti L., Qi L., Dunsdon R.M., Dymock B.W., Jones P., Overton S.H. (2003). Identification of a novel class of orally active pyrimido[5,4-*e*][1,2,4]triazine-5,7-diamine-based hypoglycemic agents with protein tyrosine phosphatase inhibitory activity. Bioorg. Med. Chem. Lett..

[9] Sugimoto T., Matsuura S. (1975). A new synthesis of pyrimido[4,5-e][1,2,4]triazines from 5,5-dibromopyrimidines. Bull. Chem. Soc. Jpn..

[10] Mohammed F.K., Badrey M.G. (2011). Synthesis of pyrimidines and heteroannulated pyrimidine ring systems. J. Korean Chem. Soc..

[11] Gomha S.M., Abdel-Aziz H.A. (2012). Synthesis of new heterocycles derived from 3-(3-methyl-1*H-*indol-2-yl)-3-oxopropanenitrile as potent antifungal agents. Bull. Korean Chem. Soc..

[12] Gomha S.M., Khalil K.D. (2012). A Convenient ultrasound-promoted synthesis and cytotoxic activity of some new thiazole derivatives bearing a coumarin nucleus. Molecules.

[13] Gomha S.M., Riyadh S.M. (2009). Synthesis of triazolo[4,3-*b*][1,2,4,5]tetrazines and triazolo[3,4-*b*] [1,3,4]thiadiazines using chitosan as ecofriendly catalyst under microwave irradiation. ARKIVOC.

[14] Gomha S.M., Abdel-Aziz H.A. (2012). Enaminones as building blocks in heterocyclic preparations: synthesis of novel pyrazoles, pyrazolo [3,4-*d*]pyridazines, pyrazolo[1,5-*a*]pyrimidines, pyrido[2,3-*d*] pyrimidines linked to imidazo[2,1-*b*]thiazole system. Heterocycles.

[15] Shawali A.S., Sherif S.M., Farghaly T.A., Shehata M.R., Darwish M.A.A. (2008). Site selectivity synthesis and tautomerism of arylazo derivatives of pyrazolo[3,4-d]pyrimido[1,6-b][1,2,4]triazine. AFINIDAD.

[16] Shawali A.S., Farghaly T.A. (2004). Synthesis and tautomeric structure of 2-[*N*-aryl-2-oxo-2-arylethanehydrazonoyl]-6-methyl-4(3*H*)-pyrimidinones. Tetrahedron.

[17] Mosselhi M.A., Pfleiderer W. (2010). Purines. Part XVI: Syntheses, properties, and reactions of 8-aminoxanthines. Helv. Chim. Acta.

[18] Shawali A.S., Mosselhi M.A., Altablawy F.M.A., Farghaly T.A., Tawfik N.M. (2008). Synthesis and tautomeric structure of 3,7-*bis*(arylazo)-6-methyl-2-phenyl-1*H*-imidazo[1,2-*b*]pyrazoles in ground and excited states. Tetrahedron.

[19] Shawali A.S., Mosselhi M.A., Farghaly T.A., Shehata M.R., Tawfik N.M. (2008). Synthesis and tautomeric structure of 3,6-*bis*(arylazo)pyrazolo[1,5-*a*]pyrimidine-5,7(4*H*,6*H*)-diones. J. Chem. Res..

[20] Farghaly T.A., Edrees M.M., Mosselhi A.M. (2012). Synthesis, tautomeric structure and antimicrobial activity of 3-arylhydrazono-4-phenyl-[1,2,4]-triazepino[2,3-a]quinazoline-2,7(1*H*)-diones. Molecules.

[21] Shawali A.S., Farghaly T.A. (2009). Synthesis and tautomeric structure of 6-arylhydrazono 1*H*-pyrazolo[3',4':4,5]-pyrimido[1,6-b][1,2,4]triazepines. Tetrahedron.

[22] Essassi E.M., Lavergne J.P., Viallefont P., Daunis J. (1975). Recherches en série triazepine-1,2,4:1-détermination de la structure de la triazolotriazépinone obtenue par action de l'acétylacétate d'éthyle sur le diamino-3,4 triazole-1,2,4. J. Heterocycl. Chem..

[23] Hassan K.M., Ahmed R.A., Abdel-Hafez S.H., Abdel-Azim M.A. (2006). Condensed thioxocyclopentapyridine (isoquinoline)-1,2,4-azines. Phosphorus Sulfur Silicon Relat. Elem..

[24] Romano C., de la Cuesta E., Avendano C., Florencio F., Sainz-Aparicio J. (1988). Reactions of 1,2-diaminobenzimidazoles with β-dicarbonyl compounds. Tetrahedron.

[25] Shawali A.S., Sherif S.M., Farghaly T.A., Shehata M.R., Darwish M.A.A. (2007). Synthesis and tautomeric structure of the azo-coupling products of 2-methyl-7-phenylpyrimido[1,2-*b*][1,2,4]triazepine-4,9(3*H*,5*H*)-dione. J. Chem. Res..

[26] Eweiss N.F., Osman A. (1980). Synthesis of heterocycles. Part II: New routes to acetylthiadiazolines and alkylazothiazoles. J. Heterocycl. Chem..

[27] Shawali A.S., Abdelhamid A.O. (1976). Reaction of dimethylphenacylsulfonium bromide with *N*-nitrosoacetarylamides and reactions of the products with nucleophiles. Bull. Chem. Soc. Jpn..

[28] Skehan P., Storeng R., Scudiero D., Monks A., McMahon J., Vistica D., Warren J.I., Bokesch H., Kenney S., Boyd M.R. (1990). New colorimetric cytotoxicity assay for anticancer-drug screening. J. Nat. Cancer Inst..

